# Gut Microbial Dysbiosis and Male Reproductive Health: Current Insights and Future Directions

**DOI:** 10.3390/ijms27104482

**Published:** 2026-05-16

**Authors:** Melody Pan, Cristian O’Flaherty

**Affiliations:** 1 Department of Pharmacology and Therapeutics, McGill University, Montreal, QC H3G 1Y6, Canada; melody.pan@mail.mcgill.ca; 2 The Research Institute, McGill University Health Centre, Montreal, QC H3H 2R9, Canada; 3 Department of Surgery (Urology Division), McGill University, Montreal, QC H3G 1A4, Canada; 4 Department of Anatomy and Cell Biology, McGill University, Montreal, QC H3A 0C7, Canada

**Keywords:** male infertility, gut microbiome, gut microbial dysbiosis, gut–testis axis

## Abstract

Male infertility contributes to approximately half of all infertility cases, with a substantial proportion remaining idiopathic. Emerging evidence implicates the gut microbiome as a regulator of male reproductive health through a proposed gut–testis axis. Few studies have shown that gut microbial dysbiosis may impair sperm quality via multiple mechanisms, including disruption of endocrine function (e.g., reduced testosterone production), alterations in microbial-derived metabolites, and impaired testicular energy metabolism. Increased intestinal permeability and systemic inflammation may further compromise the blood–testis barrier, while translocation of bacterial endotoxins may also contribute to testicular damage. Collectively, these processes can disrupt spermatogenesis and negatively affect sperm parameters, such as concentration, motility, and morphology. Interventions that restore microbial balance, including dietary modulation, have shown potential in reversing these effects and improving reproductive outcomes. This review summarizes and evaluates current literature linking gut microbial dysbiosis to male reproductive dysfunction. Key methodological limitations and knowledge gaps are highlighted, providing a foundation for advancing the development of gut microbiome-based interventions to improve male reproductive health.

## 1. Introduction

Infertility, as defined by the World Health Organization (WHO), is the inability to conceive after one year of regular, unprotected sexual intercourse [1,2]. This condition affects approximately one in six couples worldwide, with male-related factors contributing to nearly half of all cases [2,3,4]. Couples experiencing infertility often face societal, emotional and financial challenges, highlighting this condition as a significant global health concern [5]. Despite advancements in elucidating the biological, environmental, and lifestyle factors that influence reproductive health, many male infertility cases remain unexplained. This persistent knowledge gap emphasizes the need to identify novel biological pathways that contribute to reproductive dysfunction, particularly in cases of idiopathic infertility.

Growing evidence indicates that gut microbiome-mediated processes play an important role in male reproductive health, suggesting the existence of a gut–testis axis [6,7,8,9,10,11,12,13,14,15,16]. Diet, lifestyle, and the host’s metabolic status influence the gut microbiome and may therefore be determinants of male fertility.

This review aims to summarize and critically evaluate the existing evidence linking gut microbial dysbiosis to impaired male fertility. In doing so, we highlight key knowledge gaps and limitations within the field, providing a foundation for the development of gut microbiome-based therapeutic strategies to address male infertility.

## 2. Male Infertility

Male infertility refers to abnormalities in male reproductive function that impair the ability to achieve conception [2,4]. It is a multifactorial condition arising from defects in sperm production, sperm function, or obstruction of ejaculation [4]. Epidemiological data indicate a 0.3% annual increase in the age-standardized prevalence of male infertility, highlighting a growing clinical challenge [17].

Clinically, male infertility is characterized by impaired sperm parameters, including oligozoospermia (reduced sperm concentration), azoospermia (absence of sperm), asthenozoospermia (reduced sperm motility), or teratozoospermia (abnormal sperm morphology) [18]. These classifications are defined according to the WHO manual for human semen analysis, providing standardized reference thresholds for normozoospermia based on semen volume (≥1.5 mL), sperm count (≥39 million sperm per ejaculate), motility (≥40% total motility, ≥32% progressive motility), as well as normal morphology (≥4%) [19]. Impaired male fertility is diagnosed when semen parameters fall below these reference limits as well as when functional sperm defects are present.

Standard evaluation of male infertility typically includes clinical history, physical examination, hormonal profiling, and semen analysis (spermogram) [19]. Semen analysis assesses parameters such as sperm concentration, total sperm count, motility, viability and morphology. Sperm motility and kinematics may be further evaluated using computer-assisted semen analysis (CASA) [20]. Despite its clinical utility, conventional semen analysis has limited predictive value because it does not fully capture key functional parameters, such as oxidative stress status, sperm DNA fragmentation, and the ability to undergo capacitation. Although assays such as the Sperm Chromatin Structure Assay (SCSA) and the Sperm Chromatin Dispersion (SCD) test provide additional insight into DNA fragmentation, these methods are not routinely incorporated into clinical practice [21,22,23].

Male infertility arises from diverse etiologies, including medical conditions (e.g., varicocele, cystic fibrosis, infections, and reproductive tract obstruction), genetic abnormalities (e.g., Klinefelter syndrome), hormonal dysregulation (e.g., hypogonadism), age-related diseases (e.g., hypertension, diabetes, and cancer), environmental exposures (e.g., endocrine-disrupting chemicals and heavy metals), and adverse lifestyle factors such as obesity, smoking, alcohol consumption and drug use [4,5,24,25].

However, despite these diagnostic approaches and established risk factors, a substantial proportion of cases remain idiopathic. Idiopathic male infertility is defined by the absence of an identifiable cause following comprehensive clinical evaluation, often despite normal semen parameters and exclusion of known conditions such as hormonal abnormalities, varicocele, or infection. Notably, 34% of infertile men fall into this category, highlighting the limitations of current diagnostic approaches and the presence of underlying biological mechanisms that remain poorly understood [26].

Given the sensitivity of the male reproductive system to inflammatory, metabolic, and oxidative stressors, increasing attention has focused on the potential contribution of gut microbial dysbiosis to male infertility pathogenesis.

## 3. The Gut Microbiome and Gut Microbial Dysbiosis

The human gut microbiota is a dynamic ecosystem composed of bacteria, archaea, fungi and viruses that inhabit the gastrointestinal tract [27]. This microbial community plays a fundamental role in regulating host physiology, influencing immune function [28], metabolic homeostasis [29], and endocrine signaling [30].

Within this community, gut microbiota bacteria can be broadly categorized into beneficial commensals and potentially harmful pathobionts based on their functional impact on host physiology. Beneficial microorganisms, including short-chain fatty acid (SCFA)-producing taxa such as *Bifidobacterium*, *Lactobacillus*, and *Faecalibacterium*, support host health by preventing pathogen colonization [31], maintaining intestinal epithelial integrity [32], and modulating both local and systemic immune responses [33]. In addition, commensal taxa synthesize essential micronutrients, including vitamin K and various B-group vitamins such as folate and riboflavin, which support important cellular functions [34,35]. For example, folate, produced by species such as *Bifidobacterium*, *Lactobacillus*, *Bacteroides*, and *Faecalibacterium* [36] is required for nucleic acid synthesis, genomic stability, DNA methylation and cell division [37,38,39]. Riboflavin, a product of *Lactobacillus* and *Bifidobacterium* species [40], contributes to cellular redox reactions and energy metabolism through its role as a precursor for flavin mononucleotide (FMN) and flavin adenine dinucleotide (FAD) [41]. In contrast, under conditions of host or environmental perturbation, pathobionts, particularly endotoxin-producing Gram-negative bacteria such as members of the *Proteobacteria* phylum, can expand and promote inflammation, oxidative stress, and metabolic dysfunction [42,43]. These microbes produce proinflammatory molecules such as lipopolysaccharide (LPS), whose lipid A moiety activates pattern recognition receptors such as Toll-like receptor 4 (TLR4) and myeloid differentiation factor 2 (MD2) signaling complexes on immune cells, triggering nuclear factor-κB (NF-κB) activation and downstream proinflammatory cytokine production [44,45]. Sustained activation of these pathways contributes to chronic inflammation and has been implicated in disorders such as inflammatory bowel disease and Crohn’s disease [42,43].

A key metabolic role of the gut microbiota is the fermentation of indigestible dietary polysaccharides into SCFAs, such as acetate, propionate, and butyrate. These metabolites maintain the barrier function of the intestinal tract, act as bioactive molecules in energy metabolism, regulate immune cell development and exert anti-inflammatory effects [46,47]. Among SCFAs, butyrate is particularly important in maintaining colonic epithelial health by enhancing tight junction protein expression, thus guaranteeing gut epithelial barrier integrity [48], and by regulating histone deacetylase activity, thereby influencing gene expression and anti-inflammatory signaling pathways [49,50,51]. The gut microbiome also metabolizes host-derived compounds, namely primary bile acids and steroid hormones, into secondary metabolites that function as signaling molecules [52]. Thus, it further influences lipid metabolism, glucose homeostasis, immune cell differentiation, and endocrine signaling. Therefore, disruptions in gut microbial composition can lead to dysbiosis and exert deleterious effects on the host’s health.

Gut microbial dysbiosis refers to a state of microbial imbalance characterized by a loss of beneficial bacteria, an overgrowth of pathogenic bacteria, and a reduction in microbial diversity [53]. One of the main consequences of gut microbial dysbiosis is increased intestinal permeability or “leaky gut”, leading to the translocation of bacterial metabolites and endotoxins, such as LPS, into the systemic circulation [54]. This barrier dysfunction is often driven by a reduction in butyrate-producing bacteria, such as the anaerobic Gram-positive bacterium from the *Firmicutes* phylum, leading to a decreased expression of tight junction proteins [50,54]. And so, the systemic circulation of LPS and other microbial products can trigger systemic inflammation and metabolic endotoxemia, which, in turn, promotes excessive reactive oxygen species (ROS) generation, propagating pathophysiological effects far beyond the gastrointestinal tract [54]. As a result, gut microbial dysbiosis has been implicated in the pathogenesis of numerous diseases, including inflammatory bowel disease [55], metabolic dysfunction-associated steatohepatitis [56], obesity [57], diabetes [58], and neurodegeneration [59].

Importantly, in the context of male reproductive health, gut microbial dysbiosis is proposed to disrupt hormonal regulation, metabolic homeostasis, and inflammatory singalling, collectively impairing spermatogenesis and sperm function. Consistent with this model, preclinical studies demonstrate an association between gut microbial dysbiosis-induced inflammation with endocrine and metabolic disturbances, leading to increased oxidative stress and impaired spermatogenesis [6,7,8,9,10,11]. Conversely, dietary or pharmacological interventions that restore gut microbial balance are correlated with improved semen quality [7,12,13,14,15,16,60,61]. These findings highlight the gut–testis axis as a promising therapeutic target; however, evidence in human studies remains largely correlative, with limited mechanistic validation.

## 4. Investigating the Role of Gut Microbial Dysbiosis in Male Infertility

### 4.1. Gut Microbiota-Derived Factors Influencing Male Reproductive Health

Several studies have begun to investigate the role of gut microbial-derived metabolites in male reproductive health, as summarized in Table 1. Although current evidence linking specific metabolite classes to male reproductive function remains limited, microbial metabolites such as SCFAs and amino acid-derived compounds have been shown to influence testicular development, endocrine signalling, blood–testis barrier (BTB) integrity, and oxidative stress. In parallel, non-metabolic microbial components, including bacterial endotoxins, have emerged as important mediators of reproductive dysfunction, impairing semen quality through inflammatory and oxidative stress-dependent mechanisms.

#### 4.1.1. Short-Chain Fatty Acids (SCFAs) in Testicular Function and Spermatogenesis

SCFAs, particularly butyrate, are well-characterized gut microbial metabolites implicated in male reproductive health. Evidence from germ-free (GF) mouse models has demonstrated that the absence of a gut microbiota is associated with impaired testicular development [6]. More specifically, GF mice exhibited lower serum levels of gonadotropins (FSH and LH), which are critical for maintaining spermatogenesis, as well as delayed seminiferous tubule lumen formation. Accordingly, GF mice also display increased BTB permeability, characterized by reduced expression of tight junction proteins, including Occludin and E-Cadherin, compared with conventionally colonized mice. This disruption of the BTB compromises the testis’s immune-privileged environment, allowing immune cell infiltration that further disrupts spermatogenesis and impairs sperm quality [62]. Notably, colonization of GF mice with *Clostridium Tyrobutyricum,* a high butyrate-producing bacterium, restored BTB function and normalized the expression of adhesion proteins, suggesting the role of SCFAs in maintaining testicular structure and function [6]. Given the importance of proper seminiferous tubule lumen formation in Sertoli cell differentiation [63], these findings further suggest that microbiota-derived metabolites may regulate Sertoli cell maturation and, consequently, BTB integrity. SCFAs, such as butyrate, have also been shown to increase LH [64] and FSH [65] levels, indicating that the absence of these beneficial SCFA-producing taxa may contribute to the reduced gonadotropin levels observed in GF mice [6].

Complementary findings from a dietary intervention study reinforce these observations. Supplementation with high-fibre diets, probiotics, or tributyrin (a butyric acid precursor) in young boars before sexual maturity has been shown to increase the abundance of beneficial SCFA-producing bacteria such as *Rumenococcus* (phylum *Bacillota*) and *Lactobacillus* (phylum *Bacillota*) [7]. These shifts in microbial composition were associated with elevated total SCFA levels and improved sperm motility and viability [7]. These effects may be partially attributed to butyric acid’s role in enhancing antioxidant capacity within the testes [66], as well as its epigenetic regulatory function as a histone deacetylase (HDAC) inhibitor, through which it modulates gene expression involved in spermatogenesis [67,68]. Collectively, these findings highlight SCFAs as key mediators of gut microbiota-driven regulation of testicular function.

#### 4.1.2. Gut Microbiota-Regulated Amino Acid Metabolism in Testicular Function

In addition to SCFAs, amino acid-derived metabolites have also been shown to play a role in maintaining testicular homeostasis. Shan et al. [8] demonstrated that a high-concentrate diet induces gut microbial dysbiosis in mice, resulting in reduced circulating levels of L-citrulline, a precursor to arginine and a key metabolite in nitric oxide (NO) synthesis. Although L-citrulline is primarily synthesized endogenously by enterocytes, its systemic availability is closely linked to intestinal health and microbial composition. Gut microbial dysbiosis can impair enterocyte function and mass, alter intestinal metabolism, and disrupt host–microbe metabolic cross-talk, thereby reducing L-citrulline production and availability [69]. NO is essential for regulating Sertoli cell–germ cell interactions and maintaining BTB dynamics by modulating junctional protein expression and barrier permeability [70,71]. Consequently, reduced L-citrulline levels may compromise these functions.

Additionally, L-citrulline contributes to systemic antioxidant defenses as a precursor of arginine [72]; thus, its depletion may exacerbate oxidative stress and DNA damage within Sertoli cells. Consistent with this, impaired Sertoli cell function was observed, including increased apoptosis, disrupted BTB integrity, and abnormal seminiferous tubule morphology. Importantly, dietary supplementation with L-citrulline improved these defects, likely by enhancing antioxidant capacity and thereby improving sperm quality. Correlation analyses further revealed strong associations between L-citrulline and DNA repair markers, including *Pcna* and *Gtf2h5*, suggesting that supplementation with this gut-derived metabolite may also play a role in activating DNA repair mechanisms in Sertoli cells.

Together, these studies support the concept that microbial-derived amino acid metabolites influence testicular development, BTB integrity and sperm quality; however, in contrast to these regulatory effects, gut microbial dysbiosis can also generate bacterial-derived toxins that exert detrimental effects on male reproductive function.

#### 4.1.3. Bacterial Endotoxins and Impaired Sperm Quality

A growing body of evidence demonstrates that bacterial endotoxins directly impair both sperm and testicular function. Multiple cell types, including Sertoli, Leydig, and spermatozoa, have been shown to express members of the TLR family, enabling direct responsiveness to bacterial endotoxins [73,74,75,76].

In vitro studies using isolated Sertoli cells show that LPS exposure disrupts key supportive functions required for spermatogenesis, including reduced plasminogen activator activity, alongside increased ROS generation [75,76]. These alterations are accompanied by increased production of proinflammatory mediators, including IL-6 and IL-1 [76,77]. Consistent with these findings, in vivo administration of LPS in murine studies impairs Leydig cell function, resulting in reduced serum testosterone and luteinizing hormone (LH) levels, along with increased testicular inflammation, as evidenced by elevated IL-1β [78,79]. As testosterone, the primary androgen regulating male reproductive function, is produced by Leydig cells under LH stimulation via the hypothalamic–pituitary–gonadal (HPG) axis and is essential for the initiation and maintenance of spermatogenesis, as well as the structural and functional integrity of the seminiferous epithelium [80], its reduction has significant downstream consequences, often resulting in hypogonadism, a recognized contributor to male infertility [81]. Overall, this highlights the negative impact on bacterial endotoxins on testicular function.

Additionally, both murine and human studies further demonstrate that bacterial endotoxins, including LPS and peptidoglycan, can exert deleterious effects on mature spermatozoa by interacting with TLR4 and TLR2 expressed on the acrosomal and tail regions [74,82,83]. Exposure of sperm to LPS reduces motility and viability, induces sperm apoptosis, and impairs fertilization competence in a TLR-dependent manner [74,82,83]. These effects were largely associated with increased ROS production and elevated markers of oxidative stress, including lipid peroxidation and protein oxidation [82]. TLR activation further propagates inflammatory and apoptotic signalling through MyD88- and NF-κB-dependent pathways [84,85]. Notably, inhibition of LPS activity using polymyxin B, an antibiotic, preserves LPS-treated sperm motility and viability, further supporting a role for bacterial endotoxin-mediated TLR activation in sperm dysfunction [74].

Collectively, these findings establish that bacterial endotoxins act as a mediator linking gut microbiota dysbiosis to impaired sperm quality.

### 4.2. Fecal Microbiota Transplantation Studies on the Gut–Testis Axis

To explore the causal link in the proposed gut–testis axis, a few fecal microbiota transplantation (FMT) studies have transferred dysbiotic gut microbial communities from different etiological models into healthy animal recipients (Table 1). FMT studies across high-fat diet (HFD), high-energy diet (HED), and alcohol-induced models demonstrate that dysbiotic gut microbiota are sufficient to impair male reproductive function.

#### 4.2.1. FMT from High-Fat Diet-Induced Gut Microbial Dysbiosis

HFD-induced obesity is a well-established model of metabolic dysregulation and gut microbial dysbiosis. Ding et al. [9] demonstrated that mice fed a high-fat diet (HFD) not only resulted in gut microbial dysbiosis, characterized by an increased *Bacillota/Bacteroidota* (B/B) ratio, but also exhibited a decrease in sperm motility compared to control diet-fed mice. Importantly, FMT from HFD mice into control recipients was sufficient to transfer both microbial and reproductive phenotypes.

Recipient mice exhibited an increased abundance of *Bacteroides* and *Prevotella* (phylum *Bacteroidota*). These bacteria have been associated with proinflammatory activity, including the production of LPS and other metabolites that promote systemic inflammation and metabolic dysfunction [86,87]. Notably, elevated levels of *Prevotella copri* (*P. copri*) can exacerbate inflammation through over-fermentation, leading to the accumulation of succinate and fumarate, metabolites known to activate proinflammatory Th17 responses [88,89]. Consistent with this, a strong negative correlation was observed between sperm motility and *P. copri* abundance, with lower levels associated with improved motility. These changes were further accompanied by increased intestinal T lymphocyte infiltration and higher expression of proinflammatory cytokines in the epididymis, such as IL-1β, C-X-C motif chemokine (CXCL)-10 and monocyte chemoattractant protein (MCP)-1, which are key mediators of immune cell recruitment and amplification of inflammatory responses [90]. Collectively, these alterations coincided with impaired spermatogenesis and sperm quality. Reduced expression of genes associated with mitochondrial function in the testes was further observed, specifically those encoding subunits of mitochondrial complex I, including MT-ND1, MT-ND2, and MT-ND4, suggesting impaired oxidative phosphorylation and reduced ATP production [91]. Overall, these findings indicate that gut microbial dysbiosis may impair male reproductive function through inflammation-associated mitochondrial dysfunction and oxidative stress.

#### 4.2.2. FMT from High-Energy Diet-Induced Metabolic Syndrome

HED is commonly used to induce metabolic syndrome (MetS) and is associated with gut microbiota dysbiosis. In a study using rams with MetS induced by HED, a reduction of SCFA-producing bacteria known to promote gut barrier integrity and gut homeostasis, particularly *Ruminococcaceae NK4A214* (phylum Bacillota), was observed [10]. This microbial depletion was strongly correlated with decreased bile acid levels and impaired intestinal absorption of vitamin A, a fat-soluble vitamin whose uptake depends on bile-acid-mediated lipid digestion [92]. Given that vitamin A serves as a precursor to retinoic acid, a key regulator of meiotic initiation and spermatogonial differentiation during spermatogenesis, disruption of this pathway may directly impair germ cell development [93,94]. Consistently, HED-fed rams exhibited abnormal seminiferous tubule morphology and reduced germ cell differentiation. Importantly, when fecal microbiota from HED-fed rams were transplanted into control diet-fed mice, the recipient mice recapitulated the impaired microbial, metabolic, and reproductive phenotypes observed in the MetS model. This thereby indicates the role of a dysbiotic gut microbiota in impairing male reproductive function.

#### 4.2.3. FMT from Alcohol-Induced Metabolic Stress

Chronic alcohol consumption is another model often used to induce gut microbial dysbiosis and metabolic stress. Li et al. [11] demonstrated that FMT from alcohol-exposed mice (alcohol-FMT) into healthy recipients resulted in significantly reduced sperm quality compared to control-FMT recipients. The resulting gut dysbiosis was associated with increased intestinal levels of inflammatory cytokines and T lymphocyte aggregation in the testes. The alcohol-FMT group further exhibited reductions in the expression of testis-related genes, particularly those involved in mitochondrial function, similar to what was reported by Ding et al. [9]. Interestingly, these changes were strongly correlated with a decrease in the abundance of beneficial bacteria, such as *Lachnospiraceae_NK4A136* (phylum Bacillota), which are commonly associated with healthy individuals and serve as key producers of SCFAs, particularly butyrate, thereby contributing to the maintenance of intestinal barrier integrity and immune homeostasis [95]. Li et al. further identified 105 metabolites that were differentially altered in the alcohol-FMT group, including reduced levels of lipids, amino acids, and steroids. These reductions may impair spermatogenesis and sperm quality, as they play essential roles in sperm development and function. Lipids maintain sperm membrane integrity and fluidity, which are necessary for motility, capacitation, and fertilization [96]. Amino acids are critical for protein synthesis and cellular metabolism in spermatogenic cells [97,98], while steroid metabolites regulate androgen-mediated signaling pathways, including those involved in testosterone production [99]. Therefore, reductions in these steroid metabolites can significantly compromise sperm quality. Interestingly, metabolites involved in bile acid metabolic pathways were also significantly depleted in the alcohol-FMT group, contributing to impairments in lipid and cholesterol homeostasis, which may further negatively affect spermatogenesis.

Overall, these FMT studies do provide evidence that gut microbial dysbiosis can influence male reproductive function. Systemic inflammation, metabolic disturbances, and impairments in testicular mitochondrial function accompany these effects. However, the precise mechanisms underlying these associations remain unclear, and further research is needed to fully elucidate the gut–testis axis.

### 4.3. Studies of Therapeutic Gut Microbiota Modulation on Reproductive Dysfunction

Few studies have investigated whether restoring gut microbial balance can reverse reproductive dysfunction, as summarized in Table 1. Collectively, these interventions suggest that improving microbial composition, either through microbial transfer or dietary supplementation, can restore metabolic and inflammatory homeostasis and is linked to improved reproductive outcomes.

#### 4.3.1. Alginate Oligosaccharide (AOS)-Mediated Microbiota Restoration

Among microbiota-targeted interventions, alginate oligosaccharides (AOSs), derived from brown seaweed, have been extensively studied for their anti-inflammatory and antioxidant properties and for their ability to reshape gut microbiome composition.

In a HFD mouse model, FMT from AOS-treated mice (A10-FMT) significantly improved both HFD-induced reproductive and microbial impairments [12]. Specifically, A10-FMT mice exhibited increased sperm concentration and motility, alongside restoration of gut microbial balance, including elevated *Bacteroides* levels (phylum *Bacteroidota*), a genus involved in SCFA production, and reduced *Mucispirillum* (phylum *Deferribacterota*), a mucus-associated bacterium often linked to intestinal inflammation [100]. These microbial changes were associated with improved expression of the bile acid farnesoid X receptor (FXR), a key regulator of cholesterol and lipid metabolism that functions with retinoid X receptor (RXR) to maintain metabolic homeostasis. Increased retinol levels in the blood, liver and testes were also observed, accompanied by upregulated expression of proteins involved in retinol synthesis and storage, such as retinol dehydrogenase 9 (DHRS9) and retinol-binding protein 4 (RBP4). Retinol is essential for regulating germ cell development and meiosis during spermatogenesis [101,102], and its observed increase was associated with the restoration of spermatogenesis following A10-FMT treatment. A10-FMT mice further exhibited enhanced liver function and promoted the production of n-3 polyunsaturated fatty acids (PUFAs), including eicosapentaenoic acid (EPA) and docosahexaenoic acid (DHA), which support gut homeostasis through anti-inflammatory effects [103]. EPA and DHA are also involved in enhancing spermatogenesis, sperm membrane fluidity, motility and antioxidant capacity [104,105,106]. In the testes, these PUFAs can interact with retinoid signaling, thereby further supporting germ cell development. Therefore, given that bile acid receptors, retinol, and n-3 polyunsaturated fatty acids play essential interconnected roles in regulating lipid metabolism, hormone signaling, and germ cell development, their collective upregulation is likely to contribute to the observed restoration of spermatogenesis in A10-FMT mice.

Complementing these findings, the same authors observed similar benefits of AOS-mediated modulation of the microbiota in a type 1 diabetes (T1D) mouse model, a condition closely associated with gut microbial dysbiosis [13]. FMT from AOS-treated donors improved gut microbial composition in T1D mice by increasing *Lactobacillus* (phylum Bacillota). This beneficial bacterium supports gut barrier integrity, immune regulation, and inhibits pathogen growth through the production of SCFAs, lactic acid, and secretory immunoglobulin A (SIgA) [107], while reducing *Mycoplasma* (phylum Mycoplasmatota), a genus associated with immune dysregulation and proinflammatory cytokine production such as IL-6, IL-1β, and TNF-α [108]. Reduced blood glucose levels and improvements in spermatogenesis and sperm quality further accompanied these microbial shifts. Similar to their previous study [12], an increase in n-3 polyunsaturated fatty acids, specifically DHA and EPA, was observed in A10-FMT mice, which may contribute to the restoration of sperm impairments. This may also mitigate diabetes-associated oxidative stress by enhancing total antioxidant capacity in the blood and increasing testicular antioxidant-related proteins, including superoxide dismutase 1 (SOD1), glutathione peroxidase 1 (GPX1), and heat shock protein 70 (HSP70).

FMT from AOS-treated mice into busulfan-treated mice, a chemotherapeutic alkylating agent known to disrupt the gut microbiota and impair spermatogenesis through germ cell depletion, BTB disruption, testicular inflammation, and oxidative stress [109,110,111], was further able to rescue spermatogenesis and significantly increase sperm motility [14]. This intervention also increased the abundance of *Bacteroidales* (phylum *Bacteroidota*) and *Bifidobacteriales* (phylum *Actinobacteriota*). These beneficial bacteria are SCFA producers that strengthen gut barrier integrity and modulate systemic inflammation [112,113], which may have contributed to the reduced testicular inflammation observed in the A10-FMT mice and, consequently, to the mitigation of oxidative stress as evidenced by the increased expression of antioxidant enzyme glutathione peroxidase 1 (GPX1). These findings paralleled those observed with direct AOS treatment to busulfan-exposed mice [60].

#### 4.3.2. Fecal Microbiota Transplantation and Dietary Fibre-Driven Microbiota Restoration

Beyond AOS, direct microbial transfer and dietary modulation have also been shown to restore reproductive function. In the HFD-induced obese mouse model, both treatment with a high-fibre diet and FMT from control mouse donors (control-FMT) significantly improved reproductive outcomes [15]. HFD-induced obesity was associated with testicular dysfunction, including disrupted seminiferous tubule structure, increased germ cell apoptosis, reduced testosterone levels, and impaired spermatogenesis. These changes were accompanied by gut microbiota dysbiosis, with reductions in beneficial taxa such as *Lachnospiraceae_NK4A136* (phylum *Bacillota*), a key SCFA-producing bacterium. Depletion of SCFA producers may compromise gut barrier integrity and trigger systemic inflammation [95], thereby activating testicular inflammatory pathways. Consistent with these findings, HFD mice exhibited increased expression of the NLRP3/ASC/caspase-1 axis. This multiprotein inflammasome complex promotes the release of proinflammatory cytokines, such as IL-β, which can then induce germ cell apoptosis and other observed reproductive impairments as previously mentioned.

In contrast, control-FMT and supplementation with a high-fibre diet improved testicular morphology and function. These interventions restored a microbial composition resembling that of control-diet-fed mice by increasing SCFA-producing taxa, such as *Lachnospiraceae_NK4A136* and *Lactobacillaceae.* Additionally, metabolomic analyses revealed enrichment of steroid-related metabolites following treatment, including cortisol 21-acetate and hydrocortisone. These metabolites have anti-inflammatory effects that can inhibit IL-1β-induced inflammatory responses [114], potentially reducing germ cell apoptosis as observed in FMT or high-fibre diet-fed mice.

Aging-related declines in male fertility have also been linked to gut microbiota dysbiosis, and FMT has emerged as a potential intervention in this context [16]. Fecal microbiota from young mouse donors were transplanted into aging mice, and significant improvements in spermatogenesis and sperm motility were observed in the transplanted group. Age-associated gut microbial dysbiosis was characterized by an increase in LPS-producing bacteria, such as Enterobacteriaceae (phylum *Pseudomonadota*), which promote inflammation, and a decrease in Bacteroidetes, a SCFA producer. FMT from young mice reversed these microbial changes and increased 3-hydroxyphenylacetic acid (3-HPAA) production, thereby enhancing GPX4 expression. 3-HPAA is a major intestinal catabolite of quercetin glycosides and possesses antioxidant and anti-apoptosis effects [115], while GPX4 protects cells from oxidative damage [116]. As a result, this pathway was associated with lower levels of lipid peroxidation in the testis, thereby facilitating improved spermatogenesis in FMT-treated old mice.

Taken together, these studies indicate the therapeutic potential of modulating the gut microbiota to reverse male reproductive impairments. Restoration of SCFA-producing taxa, suppression of inflammasome-mediated inflammation, and enhancement of antioxidant defences emerge as central drivers of improved reproductive function. These microbial shifts are further linked to systemic metabolic reprogramming, including bile acid, retinoid, lipid, and steroid metabolism, which together support spermatogenesis and sperm function.

### 4.4. Emerging Clinical Evidence from Human and Translational Studies

Compared with animal studies, human evidence linking gut microbial dysbiosis to male infertility remains limited and largely derives from cross-sectional studies, as summarized in Table 2. These studies suggest that host metabolic status and dietary patterns may influence reproductive outcomes by modulating inflammation, hormonal balance, and sperm function.

#### 4.4.1. Metabolic Disease-Associated Gut Microbial Dysbiosis in Type 2 Diabetes

Men with type 2 diabetes (T2D) and testosterone deficiency exhibited a different gut microbiota composition compared to those with T2D alone, characterized by an increased abundance of opportunistic pathogens [117]. More specifically, proinflammatory taxa like *Parvimonas* (phylum Bacillota)*, Actinomyces* (phylum Actinobacteria), along with LPS-producing gram-negative bacteria, including *Bergeyella* (phylum Bacteroidetes) and *Massilia* (phylum Pseudomonadota), were enriched in the testosterone-deficient individuals [118,119,120]. In addition, bacteria such as *Lachnoclostridium, Blautia, and Bergeyella* showed positive correlations with insulin resistance markers (e.g., HOMA-IR) and negative associations with circulating testosterone levels. *Lachnoclostridium* and *Streptococcus* were also negatively correlated with gonadotropins such as FSH and LH, suggesting a potential role for gut microbiota in modulating testosterone production via the hypothalamic–pituitary–gonadal (HPG) axis. Overall, the enrichment of these proinflammatory and LPS-producing bacteria promotes systemic inflammation and oxidative stress, which are factors known to impair and disrupt the HPG axis, resulting in reduced testosterone production and altered gonadotropin release needed for proper spermatogenesis [80]. Consequently, these microbial changes may contribute to impaired sperm quality.

In contrast, men with T2DM alone harboured gut microbes that support intestinal homeostasis, such as *Candidatus Saccharimonas*, which help mitigate inflammation and preserve gut barrier integrity [121]. Observational data from elderly male populations without prostate cancer have further revealed a positive correlation between the phylum Bacillota and circulating testosterone levels, independent of age, BMI, and lipid profiles [122]. These findings suggest that gut microbial composition may influence androgen status even in the absence of overt metabolic diseases.

**Table 2:** Summary of clinical studies investigating the gut–testis axis.

Study	Model	Main Findings
Liu et al. [117]	Observational study comparing gut microbiota composition in men with T2DM vs. those with T2DM and testosterone deficiency	Men with T2DM and testosterone deficiency exhibit altered gut microbiota composition, characterized by an increased abundance of opportunistic and proinflammatory taxa, which are associated with metabolic dysfunction and insulin resistance.
Matsushita et al. [122]	Observational study examining gut microbiota composition and testosterone levels in elderly men	Positive correlation between the phylum Bacillota and circulating testosterone levels
Liu et al. [123]	Cross-sectional study of Taiwanese men examining the association between different dietary patterns and semen quality	High intake of “Western diet” and “ High sweet snacks & sugar sweetened drinks” were associated with impairments in sperm quality
Jensen et al. [124]	Cross-sectional study examining the association between dietary fat intake and semen quality in Danish men	Lower sperm concentration and total sperm count in men with a high intake of saturated fat
Attaman et al. [125]	A cross-sectional study observing the correlation between high intake of saturated fats and sperm quality	Higher intake of total fat was negatively correlated with total sperm count and concentration
Mendiola et al. [126]	Observational study comparing dietary habits in normospermic and oligoasthenoteratospermic patients	Higher intake of dairy and processed meat products was associated with greater impairments in sperm quality
Wang et al. [127]	Mendelian randomization study for gut microbiota and male fertility	Ruminiclostridium6, Prevotella9, and members of the Lachnospiraceae family are associated with male infertility
Cao et al. [128]	Examining differences in the intestinal microbiota between healthy individuals and those with abnormal sperm parameters	Reductions in *Bifidobacterium*, *Collinsella*, and *Blautia* were observed in men with abnormal sperm parameters

#### 4.4.2. Dietary Patterns and Western-Style Diets

Notably, across multiple studies, a Western-style diet, characterized by high intake of saturated fats, refined carbohydrates, and sugar-sweetened foods, has been consistently associated with poor semen parameters [123,124,125].

In an Asian male cohort, dietary patterns including “Western diet” and “highly sweet snacks and sugar-sweetened drinks” were both negatively correlated with semen quality [123]. Specifically, higher intake of a Western diet, characterized by excessive dairy, meat, and fried foods, was associated with reduced sperm concentration and abnormal sperm morphology. These findings may be partially explained by increased exposure to lipophilic endocrine-disrupting compounds, such as xenoestrogens, which accumulate in fat-rich animal products and have been implicated in reproductive dysfunction [126]. In addition, high consumption of sugary foods was associated with lower sperm concentration. Increased intake of sugar has been associated with insulin resistance [1], which in turn can promote oxidative stress [129] and negatively affect sperm quality [130]; however, insulin resistance was not assessed in this study.

Several large observational studies have also examined the role of dietary fat intake in male fertility [124]. In a cohort of young Danish men attending fertility clinics, higher intake of saturated fat was strongly associated with abnormal sperm morphology, along with reduced sperm concentration and total sperm count [125]. Similarly, other studies have reported that a higher intake of saturated fats, meat and dairy products, which may carry high levels of xenoestrogens and disrupt endocrine function, was correlated with poor sperm count and quality within men attending fertility clinics for reproductive assistance [126]. Conversely, higher intake of fruits and vegetables, rich in antioxidants and micronutrients, was associated with improved semen quality, suggesting a protective role in mitigating oxidative stress [126]. Higher intake of omega-3 polyunsaturated fatty acids was also positively associated with improved sperm morphology and overall semen quality [125]. Omega-3 PUFAs may exert protective effects through antioxidant, anti-inflammatory and anti-apoptotic effects, thus contributing to the observed changes [131,132].

#### 4.4.3. Mendelian Randomization Evidence Linking Gut Microbiota and Male Fertility

**Figure 1:**
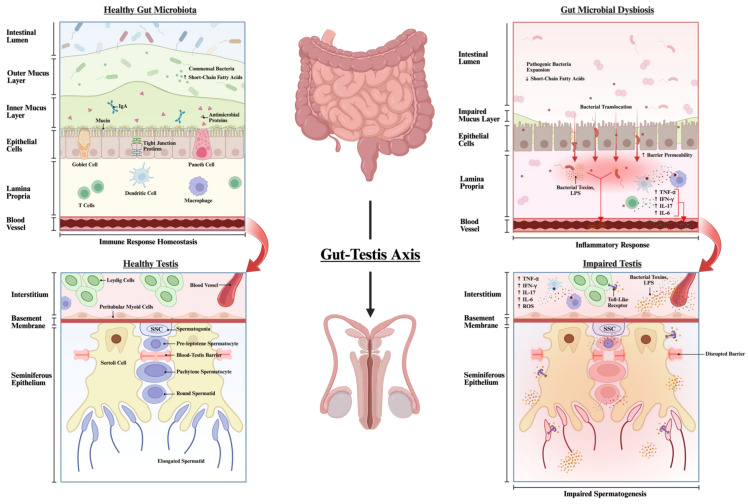
Overview of the proposed gut–testis axis in a healthy gut microbiota compared to gut microbial dysbiosis. Schematic representation of the relationship between the gut microbiota and testicular function under physiological and dysbiotic conditions. In a healthy state (**left**), a diverse and balanced gut microbiota supports intestinal barrier integrity by producing beneficial metabolites (e.g., short-chain fatty acids), maintaining a structured mucus layer, and regulating immune signalling. This homeostatic environment limits bacterial translocation and systemic inflammation, thereby preserving blood–testis barrier integrity and supporting normal spermatogenesis within the seminiferous epithelium. In contrast (**right**), gut microbial dysbiosis is characterized by reduced beneficial metabolites, expansion of pathogenic bacteria, and impaired mucus barrier function, leading to increased intestinal permeability (“leaky gut”). Translocation of bacterial components, such as lipopolysaccharide (LPS), into the circulation triggers systemic inflammation characterized by elevated proinflammatory cytokines (e.g., TNF-α, IFN-γ, IL-6, IL-17). LPS can directly activate Toll-like receptors (TLR) expressed on Leydig cells, Sertoli cells, and spermatozoa, initiating local inflammatory and oxidative stress responses within the testes. These signals disrupt the blood–testis barrier, impair Sertoli and germ cell function, and ultimately compromise spermatogenesis. Created in BioRender, https://www.biorender.com/, Pan, M. (2026) (accessed on 1 May 2026).

Beyond observational dietary associations, Mendelian randomization analysis has been used to explore potential causal links between the gut microbiota and male fertility. This approach identified an association of *Ruminiclostridium* (phylum Bacillota) and *Prevotella* (phylum Bacteroidota) with male infertility, including abnormal sperm parameters and erectile dysfunction [1]. *Ruminiclostridium* has been linked to gut microbial dysbiosis and metabolic disorders, partly due to its association with increased production of proinflammatory cytokines such as IL-6, IL-1β and TNF-α [133,134]. Similarly, *Prevotella* can promote inflammation by driving excessive fermentation and activating proinflammatory Th17 responses [88,89]. Collectively, the increase in these taxa may contribute to a proinflammatory environment that impairs spermatogenesis. Consistent with these findings, the study by Ding et al. also reported a strong association between elevated *Prevotella* abundance in the gut microbiota and impaired spermatogenesis [9] (Figure 1).

A small cohort study further reported altered gut microbial composition in men with impaired semen quality [128]. Interestingly, reductions in beneficial SCFA-producing bacteria, including *Bifidobacterium* (phylum Actinobacteria), were associated with decreased sperm motility. *Bifidobacterium* supports gut barrier integrity, inhibits proinflammatory cytokines [135], produces metabolites such as γ-aminobutyric acid (GABA), which promotes the acrosome reaction [136], and facilitates sperm capacitation through increased tyrosine phosphorylation of the sperm proteins [137]. In addition, reduced abundance of *Blautia* (phylum Bacillota) was also associated with decreased sperm motility. *Blautia* is a beneficial bacterium that can inhibit pathogenic bacterial colonization in the gut by producing bacteriocins [138]. Altogether, the depletion of these beneficial taxa may contribute to impaired sperm quality by increasing systemic inflammation and reducing the availability of metabolites that support normal sperm function.

Overall, these studies show an association between gut microbial composition, dietary patterns, and male reproductive outcomes. Any alterations in the gut microbiota profile are correlated with hormonal disturbances and reduced sperm quality. However, these studies are limited by numerous uncontrolled confounding factors, a lack of longitudinal studies, and their inability to establish causality.

## 5. Limitations and Current Knowledge Gaps

Despite the growing evidence on the potential role of the gut microbiome in male reproductive function, several critical knowledge gaps persist in the current literature.

One limitation is the incomplete understanding of the specific mechanisms underlying the gut–testis axis. The majority of published studies are either observational or cross-sectional. While they are valuable for identifying associations between dietary patterns, gut microbial dysbiosis, and impaired sperm parameters, these studies are limited in their ability to establish causality. More specifically, they lack elucidation of the mechanistic pathways through which microbial alterations influence key testicular processes, including Leydig cell steroidogenesis, Sertoli cell support of germ cells, maintenance of the BTB, and overall spermatogenesis.

Consequently, the biological significance of many microbial taxa, particularly those of low abundance or closely related species, remains poorly characterized [139]. Moreover, while gut microbiota-derived metabolites are known to influence host physiology, their absorption, distribution and metabolism in the context of male reproductive organs are poorly defined. It remains unclear how these metabolites reach and act upon the testicular environment, the concentrations at which they exert biological effects, and the signaling pathways through which they influence spermatogenesis. A future investigation would be to integrate multi-omics approaches to provide a comprehensive understanding of the gut microbiome’s structure and function, specifically under dysbiotic conditions.

The heterogeneity in experimental designs further complicates the interpretation of the gut–testis axis. In animal models, diet- and disease-induced gut microbial dysbiosis varies widely in composition, duration, and severity. Similarly, dietary intervention protocols within human cross-sectional studies are often inconsistent in design and lack appropriate control groups. Numerous confounding factors, including age, genetics, body mass index (BMI), lifestyle habits, medication use, and demographic differences, can all independently influence both gut microbial composition and reproductive outcomes [140,141]. In addition, the lack of large-scale, longitudinal studies to track microbial changes over time further limits understanding of the gut microbiome’s causal role in male infertility. Another major challenge lies in the lack of standardized protocols for microbiome profiling. Variability in sample collection, DNA extraction methods, and sequencing platforms introduces inconsistencies in microbial identification and taxonomic resolution across studies, thereby limiting reproducibility.

## 6. Conclusions

Collectively, current evidence suggests that the gut microbiome plays a critical role in male reproductive health, with gut microbial dysbiosis increasingly linked to male infertility. Key animal models demonstrate that gut microbial dysbiosis promotes systemic inflammation, alters gut microbial-derived metabolites, and compromises intestinal epithelial integrity, all of which are associated with disrupted spermatogenesis and impaired sperm parameters. Restoration of the gut microbiome through dietary interventions, probiotics, and other microbiome-modulating strategies has been shown to rescue these impairments. Despite these findings, there remains a lack of targeted mechanistic studies to fully understand the gut–testis axis.

Continued advancement in this field will aid not only in identifying specific microbial targets but also in examining how gut microbes interact with the host. Overall, this review highlights gaps in the current literature and the need for further research before leveraging the gut microbiome as a promising frontier for managing impaired male reproductive health.

## Figures and Tables

**Table 1:** Summary of key animal studies investigating the gut–testis axis.

Study	Model	Main Findings
Al-Asmakh et al. [6]	GF mice and GF mice colonized with *C. tyrobutyricum*	Delayed seminiferous tubule lumen formation and increased BTB permeability in GF mice. Colonization with *C. tyrobutyricum* restored BTB structure and tight junction protein expression
Lin et al. [7]	Boars fed a control diet and a high-fibre diet, with or without tributyrin supplementation	A high-fibre diet and tributyrin supplementation elevated SCFAs abundance and improved sperm viability
Shan et al. [8]	HCD-fed sheep and HCD-fed mouse model, then supplemented with L-citrulline	L-citrulline supplementation restores Sertoli cell damage and BTB integrity induced by HCD
Ding et al. [9]	FMT from HFD-fed mice to control mice recipients	HFD-FMT mice exhibited impaired sperm quality, increased abundance of *Bacteroidetes* and *Prevotella*, and elevated proinflammatory cytokines in the epididymis
Zhang et al. [10]	Sheep model of MetS induced by HED and HED-FMT mouse model	HFD-FMT mice model had impaired vitamin A metabolism, reduced bile acids, and impaired spermatogenesis compared to control mice
Li et al. [11]	FMT from alcohol-fed mice to control mice	Alcohol-FMT mice exhibited impaired sperm quality, increased intestinal inflammatory cytokines, and decreased abundance in *Lachnospiraceae_NK4A136* compared to control mice
Hao et al. [12]	FMT from AOS-treated mice into the HFD-fed mouse model	AOS-FMT mice restored gut microbiota and improved sperm quality and liver function
Hao et al. [13]	FMT from AOS-treated mice into T1D mouse model	AOS-FMT restored gut microbial dysbiosis induced by T1D, reduced blood glucose levels and improved sperm quality
Zhang et al. [14]	FMT from AOS-treated mice into a busulfan-treated mouse model	AOS-FMT improved sperm quality and increased abundances of *Bacteroidales* and *Bifidobacteriales*
Huang et al. [15]	HFD-mice treated with a high-dietary fibre diet or FMT from control donors	FMT or a high-fibre diet restored gut microbiota composition, enriched anti-inflammatory metabolites, and improved testicular function
Jin et al. [16]	FMT from young mice into old mouse models	FMT from young mice to old mice alleviated aging-associated spermatogenic dysfunction and restored gut microbiota dysbiosis

## Data Availability

No new data were created or analyzed in this study. Data sharing is not applicable to this article.

## Abbreviations

The following abbreviations are used in this manuscript:

AOS	Alginate oligosaccharides
BTB	Blood–testis barrier
CASA	Computer-assisted semen analysis
CXCL-10	C-X-C motif chemokine
FAD	Flavin adenine dinucleotide
FMN	Flavin mononucleotide
FMT	Fecal microbiota transplant
GABA	γ-Aminobutyric acid
GF	Germ-free
GPX1	Glutathione peroxidase 1
HDAC	Histone deacetylase
HED	High-energy diet
HFD	High-fat diet
HPG	Hypothalamic–pituitary–gonadal
HSP70	Heat shock protein 70
LPS	Lipopolysaccharide
MCP-1	Monocyte chemoattractant protein
MD2	Myeloid differentiation factor 2
MetS	Metabolic syndrome
NF-κB	Nuclear factor-κB
NO	Nitric oxide
ROS	Reactive oxygen species
SIgA	Secretory immunoglobulin A
SCD	Sperm chromatin dispersion
SCFAs	Short-chain fatty acids
SCSA	Sperm chromatin structure assay
SOD1	Superoxide dismutase 1
T1D	Type 1 diabetes
T2D	Type 2 diabetes
TLR	Toll-like receptor

## References

[1] Hou K., Wu Z.-X., Chen X.-Y., Wang J.-Q., Zhang D., Xiao C., Zhu D., Koya J.B., Wei L., Li J. (2022). Microbiota in health and diseases. Signal Transduct. Target. Ther..

[2] World Health Organization (2023). Infertility Prevalence Estimates: 1990–2021.

[3] Carson S.A., Kallen A.N. (2021). Diagnosis and Management of Infertility: A Review. JAMA.

[4] Schlegel P.N., Sigman M., Collura B., De Jonge C.J., Eisenberg M.L., Lamb D.J., Mulhall J.P., Niederberger C., Sandlow J.I., Sokol R.Z. (2021). Diagnosis and Treatment of Infertility in Men: AUA/ASRM Guideline Part I. J. Urol..

[5] Dierickx S., De Proost M., Huang A.Y., Ceesay S., Clarke E., Balen J. (2021). The Nairobi Summit and Reproductive Justice: Unmet Needs for People with Infertility. Am. J. Trop. Med. Hyg..

[6] Al-Asmakh M., Stukenborg J.B., Reda A., Anuar F., Strand M.L., Hedin L., Pettersson S., Söder O. (2014). The gut microbiota and developmental programming of the testis in mice. PLoS ONE.

[7] Lin Y., Wang K., Che L., Fang Z., Xu S., Feng B., Zhuo Y., Li J., Wu C., Zhang J. (2022). The Improvement of Semen Quality by Dietary Fiber Intake Is Positively Related with Gut Microbiota and SCFA in a Boar Model. Front. Microbiol..

[8] Shan L., Guo X., Hu Y., Zhou H., Meng X., Liu K., Shi L., Hu F., Liu Y., Zhang T. (2025). L-citrulline protects testicular Sertoli cell function by mitigating DNA damage via the gut-testis axis of sheep fed a high-concentrate diet. npj Biofilms Microbiomes.

[9] Ding N., Zhang X., Zhang X.D., Jing J., Liu S.S., Mu Y.P., Peng L.L., Yan Y.J., Xiao G.M., Bi X.Y. (2020). Impairment of spermatogenesis and sperm motility by the high-fat diet-induced dysbiosis of gut microbes. Gut.

[10] Zhang T., Sun P., Geng Q., Fan H., Gong Y., Hu Y., Shan L., Sun Y., Shen W., Zhou Y. (2022). Disrupted spermatogenesis in a metabolic syndrome model: The role of vitamin A metabolism in the gut-testis axis. Gut.

[11] Li H., Li N., Lu Q., Yang J., Zhao J., Zhu Q., Yi S., Fu W., Luo T., Tang J. (2022). Chronic alcohol-induced dysbiosis of the gut microbiota and gut metabolites impairs sperm quality in mice. Front. Microbiol..

[12] Hao Y., Feng Y., Yan X., Chen L., Ma X., Tang X., Zhong R., Sun Z., Agarwal M., Zhang H. (2022). Gut Microbiota-Testis Axis: FMT Mitigates High-Fat Diet-Diminished Male Fertility via Improving Systemic and Testicular Metabolome. Microbiol. Spectr..

[13] Hao Y., Feng Y., Yan X., Chen L., Zhong R., Tang X., Shen W., Sun Q., Sun Z., Ren Y. (2022). Gut microbiota-testis axis: FMT improves systemic and testicular micro-environment to increase semen quality in type 1 diabetes. Mol. Med..

[14] Zhang P., Feng Y., Li L., Ge W., Yu S., Hao Y., Shen W., Han X., Ma D., Yin S. (2021). Improvement in sperm quality and spermatogenesis following faecal microbiota transplantation from alginate oligosaccharide dosed mice. Gut.

[15] Huang H., Zhou T., He F., Wen B., Yang Y., Zhong W., Wang Q., Li J. (2024). The gut microbiota improves reproductive dysfunction in obese mice by suppressing the NLRP3/ASC/caspase-1 axis. Future Microbiol..

[16] Jin Z., Yang Y., Cao Y., Wen Q., Xi Y., Cheng J., Zhao Q., Weng J., Hong K., Jiang H. (2023). The gut metabolite 3-hydroxyphenylacetic acid rejuvenates spermatogenic dysfunction in aged mice through GPX4-mediated ferroptosis. Microbiome.

[17] Sun H., Gong T.T., Jiang Y.T., Zhang S., Zhao Y.H., Wu Q.J. (2019). Global, regional, and national prevalence and disability-adjusted life-years for infertility in 195 countries and territories, 1990-2017: Results from a global burden of disease study, 2017. Aging.

[18] Kumar N., Singh A.K. (2015). Trends of male factor infertility, an important cause of infertility: A review of literature. J. Hum. Reprod. Sci..

[19] Cooper T.G., Noonan E., von Eckardstein S., Auger J., Baker H.W.G., Behre H.M., Haugen T.B., Kruger T., Wang C., Mbizvo M.T. (2010). World Health Organization reference values for human semen characteristics. Hum. Reprod. Update.

[20] Finelli R., Leisegang K., Tumallapalli S., Henkel R., Agarwal A. (2021). The validity and reliability of computer-aided semen analyzers in performing semen analysis: A systematic review. Transl. Androl. Urol..

[21] Oehninger S., Ombelet W. (2019). Limits of current male fertility testing. Fertil. Steril..

[22] Pandruvada S., Royfman R., Shah T.A., Sindhwani P., Dupree J.M., Schon S., Avidor-Reiss T. (2021). Lack of trusted diagnostic tools for undetermined male infertility. J. Assist. Reprod. Genet..

[23] Wang C., Swerdloff R.S. (2014). Limitations of semen analysis as a test of male fertility and anticipated needs from newer tests. Fertil. Steril..

[24] Assidi M. (2022). Infertility in Men: Advances towards a Comprehensive and Integrative Strategy for Precision Theranostics. Cells.

[25] Longo V., Forleo A., Provenzano S.P., Coppola L., Zara V., Ferramosca A., Siciliano P., Capone S. (2019). Seminal VOCs Analysis Investigating Sperm Quality Decline—New Studies to Improve Male Fertility Contrasting Population Ageing. Ambient Assisted Living.

[26] Agarwal A., Parekh N., Panner Selvam M.K., Henkel R., Shah R., Homa S.T., Ramasamy R., Ko E., Tremellen K., Esteves S. (2019). Male Oxidative Stress Infertility (MOSI): Proposed Terminology and Clinical Practice Guidelines for Management of Idiopathic Male Infertility. World J. Men’s Health.

[27] Turnbaugh P.J., Ley R.E., Hamady M., Fraser-Liggett C.M., Knight R., Gordon J.I. (2007). The human microbiome project. Nature.

[28] Slack E., Hapfelmeier S., Stecher B., Velykoredko Y., Stoel M., Lawson M.A., Geuking M.B., Beutler B., Tedder T.F., Hardt W.D. (2009). Innate and adaptive immunity cooperate flexibly to maintain host-microbiota mutualism. Science.

[29] Fujisaka S., Watanabe Y., Tobe K. (2023). The gut microbiome: A core regulator of metabolism. J. Endocrinol..

[30] Pires L., Gonzalez-Paramás A.M., Heleno S.A., Calhelha R.C. (2024). Gut Microbiota as an Endocrine Organ: Unveiling Its Role in Human Physiology and Health. Appl. Sci..

[31] Lawley T.D., Walker A.W. (2013). Intestinal colonization resistance. Immunology.

[32] Okumura R., Takeda K. (2017). Roles of intestinal epithelial cells in the maintenance of gut homeostasis. Exp. Mol. Med..

[33] Zheng D., Liwinski T., Elinav E. (2020). Interaction between microbiota and immunity in health and disease. Cell Res..

[34] Magnúsdóttir S., Ravcheev D., de Crécy-Lagard V., Thiele I. (2015). Systematic genome assessment of B-vitamin biosynthesis suggests co-operation among gut microbes. Front. Genet..

[35] Pham V.T., Dold S., Rehman A., Bird J.K., Steinert R.E. (2021). Vitamins, the gut microbiome and gastrointestinal health in humans. Nutr. Res..

[36] Kok D.E., Steegenga W.T., McKay J.A. (2018). Folate and Epigenetics: Why we Should Not Forget Bacterial Biosynthesis. Epigenomics.

[37] Fox J.T., Stover P.J. (2008). Folate-mediated one-carbon metabolism. Vitam. Horm..

[38] Ducker G.S., Rabinowitz J.D. (2017). One-Carbon Metabolism in Health and Disease. Cell Metab..

[39] Moulik N.R., Kumar A., Agrawal S. (2017). Folic acid, one-carbon metabolism & childhood cancer. Indian J. Med. Res..

[40] Uebanso T., Shimohata T., Mawatari K., Takahashi A. (2020). Functional Roles of B-Vitamins in the Gut and Gut Microbiome. Mol. Nutr. Food Res..

[41] Pinto J.T., Zempleni J. (2016). Riboflavin. Adv. Nutr..

[42] Buret A.G., Motta J.P., Allain T., Ferraz J., Wallace J.L. (2019). Pathobiont release from dysbiotic gut microbiota biofilms in intestinal inflammatory diseases: A role for iron?. J. Biomed. Sci..

[43] Bharathi S., Soundara Rajan Y.A.P.A., Prakash S., Immanuel G., Ramasubburayan R. (2026). Pathobionts in the microbiome: Drivers of disease and targets for treatment. Microb. Pathog..

[44] Dauphinee S.M., Karsan A. (2006). Lipopolysaccharide signaling in endothelial cells. Lab. Investig..

[45] Carpenter S., O’Neill L.A. (2009). Recent insights into the structure of Toll-like receptors and post-translational modifications of their associated signalling proteins. Biochem. J..

[46] Morrison D.J., Preston T. (2016). Formation of short chain fatty acids by the gut microbiota and their impact on human metabolism. Gut Microbes.

[47] Richards J.L., Yap Y.A., McLeod K.H., Mackay C.R., Mariño E. (2016). Dietary metabolites and the gut microbiota: An alternative approach to control inflammatory and autoimmune diseases. Clin. Transl. Immunol..

[48] Peng L., Li Z.R., Green R.S., Holzman I.R., Lin J. (2009). Butyrate enhances the intestinal barrier by facilitating tight junction assembly via activation of AMP-activated protein kinase in Caco-2 cell monolayers. J. Nutr..

[49] Arpaia N., Campbell C., Fan X., Dikiy S., van der Veeken J., deRoos P., Liu H., Cross J.R., Pfeffer K., Coffer P.J. (2013). Metabolites produced by commensal bacteria promote peripheral regulatory T-cell generation. Nature.

[50] Schulthess J., Pandey S., Capitani M., Rue-Albrecht K.C., Arnold I., Franchini F., Chomka A., Ilott N.E., Johnston D.G.W., Pires E. (2019). The Short Chain Fatty Acid Butyrate Imprints an Antimicrobial Program in Macrophages. Immunity.

[51] Chang P.V., Hao L., Offermanns S., Medzhitov R. (2014). The microbial metabolite butyrate regulates intestinal macrophage function via histone deacetylase inhibition. Proc. Natl. Acad. Sci. USA.

[52] Ridlon J.M., Kang D.J., Hylemon P.B., Bajaj J.S. (2014). Bile acids and the gut microbiome. Curr. Opin. Gastroenterol..

[53] Acevedo-Román A., Pagán-Zayas N., Velázquez-Rivera L.I., Torres-Ventura A.C., Godoy-Vitorino F. (2024). Insights into Gut Dysbiosis: Inflammatory Diseases, Obesity, and Restoration Approaches. Int. J. Mol. Sci..

[54] Levy M., Kolodziejczyk A.A., Thaiss C.A., Elinav E. (2017). Dysbiosis and the immune system. Nat. Rev. Immunol..

[55] Serban D.E. (2015). Microbiota in Inflammatory Bowel Disease Pathogenesis and Therapy: Is It All About Diet?. Nutr. Clin. Pr..

[56] Khan A., Ding Z., Ishaq M., Bacha A.S., Khan I., Hanif A., Li W., Guo X. (2021). Understanding the Effects of Gut Microbiota Dysbiosis on Nonalcoholic Fatty Liver Disease and the Possible Probiotics Role: Recent Updates. Int. J. Biol. Sci..

[57] Indiani C., Rizzardi K.F., Castelo P.M., Ferraz L.F.C., Darrieux M., Parisotto T.M. (2018). Childhood Obesity and Firmicutes/Bacteroidetes Ratio in the Gut Microbiota: A Systematic Review. Child. Obes..

[58] Sadagopan A., Mahmoud A., Begg M., Tarhuni M., Fotso M., Gonzalez N.A., Sanivarapu R.R., Osman U., Latha Kumar A., Mohammed L. (2023). Understanding the Role of the Gut Microbiome in Diabetes and Therapeutics Targeting Leaky Gut: A Systematic Review. Cureus.

[59] Tan L.Y., Yeo X.Y., Bae H.G., Lee D.P.S., Ho R.C., Kim J.E., Jo D.G., Jung S. (2021). Association of Gut Microbiome Dysbiosis with Neurodegeneration: Can Gut Microbe-Modifying Diet Prevent or Alleviate the Symptoms of Neurodegenerative Diseases?. Life.

[60] Zhao Y., Zhang P., Ge W., Feng Y., Li L., Sun Z., Zhang H., Shen W. (2020). Alginate oligosaccharides improve germ cell development and testicular microenvironment to rescue busulfan disrupted spermatogenesis. Theranostics.

[61] Zhang Y., Hou B., Liu T., Wu Y., Wang Z. (2023). Probiotics improve polystyrene microplastics-induced male reproductive toxicity in mice by alleviating inflammatory response. Ecotoxicol. Environ. Saf..

[62] Li S.Y., Kumar S., Gu X., DeFalco T. (2024). Testicular immunity. Mol. Asp. Med..

[63] Walczak-Jedrzejowska R., Slowikowska-Hilczer J., Marchlewsk K., Oszukowska E., Kula K. (2007). During seminiferous tubule maturation testosterone and synergistic action of FSH with estradiol support germ cell survival while estradiol alone has pro-apoptotic effect. Folia Histochem. Cytobiol..

[64] Ruddon R.W., Anderson C., Meade K.S., Aldenderfer P.H., Neuwald P.D. (1979). Content of gonadotropins in cultured human malignant cells and effects of sodium butyrate treatment on gonadotropin secretion by HeLa cells. Cancer Res..

[65] Ghosh N.K., Cox R.P. (1977). Induction of human follicle-stimulating hormone in HeLa cells by sodium butyrate. Nature.

[66] Alhaj H.W., Li Z., Shan T., Dai P., Zhu P., Li Y., Alsiddig M.A., Abdelghani E., Li C. (2018). Effects of dietary sodium butyrate on reproduction in adult breeder roosters. Anim. Reprod. Sci..

[67] Parab S., Shetty O., Gaonkar R., Balasinor N., Khole V., Parte P. (2015). Correction to: HDAC6 deacetylates alpha tubulin in sperm and modulates sperm motility in Holtzman rat. Cell Tissue Res..

[68] Yin H., Kang Z., Zhang Y., Gong Y., Liu M., Xue Y., He W., Wang Y., Zhang S., Xu Q. (2021). HDAC3 controls male fertility through enzyme-independent transcriptional regulation at the meiotic exit of spermatogenesis. Nucleic Acids Res..

[69] Crenn P., Messing B., Cynober L. (2008). Citrulline as a biomarker of intestinal failure due to enterocyte mass reduction. Clin. Nutr..

[70] Lee N.P.Y., Mruk D.D., Wong C.-h., Cheng C.Y. (2005). Regulation of Sertoli-Germ Cell Adherens Junction Dynamics in the Testis via the Nitric Oxide Synthase (NOS)/cGMP/Protein Kinase G (PRKG)/β-Catenin (CATNB) Signaling Pathway: An In Vitro and In Vivo Study1. Biol. Reprod..

[71] Lee N.P., Cheng C.Y. (2004). Nitric oxide/nitric oxide synthase, spermatogenesis, and tight junction dynamics. Biol. Reprod..

[72] Zhao G., Zhao X., Song Y., Haire A., Dilixiati A., Liu Z., Zhao S., Aihemaiti A., Fu X., Wusiman A. (2022). Effect of L-citrulline supplementation on sperm characteristics and hormonal and antioxidant levels in blood and seminal plasma of rams. Reprod. Domest. Anim..

[73] Shang T., Zhang X., Wang T., Sun B., Deng T., Han D. (2011). Toll-Like Receptor-Initiated Testicular Innate Immune Responses in Mouse Leydig Cells. Endocrinology.

[74] Fujita Y., Mihara T., Okazaki T., Shitanaka M., Kushino R., Ikeda C., Negishi H., Liu Z., Richards J.S., Shimada M. (2011). Toll-like receptors (TLR) 2 and 4 on human sperm recognize bacterial endotoxins and mediate apoptosis. Hum. Reprod..

[75] Bourdon V., Defamie N., Fenichel P., Pointis G. (1999). Regulation of tissue-type plasminogen activator and its inhibitor (PAI-1) by lipopolysaccharide-induced phagocytosis in a Sertoli cell line. Exp. Cell Res..

[76] Petersen C., Fröysa B., Söder O. (2004). Endotoxin and proinflammatory cytokines modulate Sertoli cell proliferation in vitro. J. Reprod. Immunol..

[77] Wu H., Wang H., Xiong W., Chen S., Tang H., Han D. (2008). Expression Patterns and Functions of Toll-Like Receptors in Mouse Sertoli Cells. Endocrinology.

[78] Gow R.M., O’Bryan M.K., Canny B.J., Ooi G.T., Hedger M.P. (2001). Differential effects of dexamethasone treatment on lipopolysaccharide-induced testicular inflammation and reproductive hormone inhibition in adult rats. J. Endocrinol..

[79] O’Bryan M.K., Schlatt S., Phillips D.J., de Kretser D.M., Hedger M.P. (2000). Bacterial Lipopolysaccharide-Induced Inflammation Compromises Testicular Function at Multiple Levels in Vivo. Endocrinology.

[80] Grande G., Barrachina F., Soler-Ventura A., Jodar M., Mancini F., Marana R., Chiloiro S., Pontecorvi A., Oliva R., Milardi D. (2022). The Role of Testosterone in Spermatogenesis: Lessons from Proteome Profiling of Human Spermatozoa in Testosterone Deficiency. Front. Endocrinol..

[81] Munari E.V., Amer M., Amodeo A., Bollino R., Federici S., Goggi G., Giovanelli L., Persani L., Cangiano B., Bonomi M. (2023). The complications of male hypogonadism: Is it just a matter of low testosterone?. Front. Endocrinol..

[82] Sahnoun S., Sellami A., Chakroun N., Mseddi M., Attia H., Rebai T., Lassoued S. (2017). Human sperm Toll-like receptor 4 (TLR4) mediates acrosome reaction, oxidative stress markers, and sperm parameters in response to bacterial lipopolysaccharide in infertile men. J. Assist. Reprod. Genet..

[83] Hagan S., Khurana N., Chandra S., Abdel-Mageed A.B., Mondal D., Hellstrom W.J., Sikka S.C. (2015). Differential expression of novel biomarkers (TLR-2, TLR-4, COX-2, and Nrf-2) of inflammation and oxidative stress in semen of leukocytospermia patients. Andrology.

[84] Aliprantis A.O., Yang R.B., Weiss D.S., Godowski P., Zychlinsky A. (2000). The apoptotic signaling pathway activated by Toll-like receptor-2. EMBO J..

[85] Kawai T., Akira S. (2007). Signaling to NF-kappaB by Toll-like receptors. Trends Mol. Med..

[86] Wexler H.M. (2007). Bacteroides: The good, the bad, and the nitty-gritty. Clin. Microbiol. Rev..

[87] Larsen J.M. (2017). The immune response to Prevotella bacteria in chronic inflammatory disease. Immunology.

[88] Jiang L., Shang M., Yu S., Liu Y., Zhang H., Zhou Y., Wang M., Wang T., Li H., Liu Z. (2022). A high-fiber diet synergizes with Prevotella copri and exacerbates rheumatoid arthritis. Cell Mol. Immunol..

[89] Guerreiro C.S., Calado Â., Sousa J., Fonseca J.E. (2018). Diet, Microbiota, and Gut Permeability-The Unknown Triad in Rheumatoid Arthritis. Front. Med..

[90] Deshmane S.L., Kremlev S., Amini S., Sawaya B.E. (2009). Monocyte Chemoattractant Protein-1 (MCP-1): An Overview. J. Interferon Cytokine Res..

[91] DiMauro S., Schon Eric A. (2003). Mitochondrial Respiratory-Chain Diseases. N. Engl. J. Med..

[92] Harrison E.H. (2012). Mechanisms involved in the intestinal absorption of dietary vitamin A and provitamin A carotenoids. Biochim. Biophys. Acta (BBA)-Mol. Cell Biol. Lipids.

[93] van Pelt A.M., de Rooij D.G. (1991). Retinoic acid is able to reinitiate spermatogenesis in vitamin A-deficient rats and high replicate doses support the full development of spermatogenic cells. Endocrinology.

[94] Vernet N., Dennefeld C., Rochette-Egly C., Oulad-Abdelghani M., Chambon P., Ghyselinck N.B., Mark M. (2006). Retinoic acid metabolism and signaling pathways in the adult and developing mouse testis. Endocrinology.

[95] Abdugheni R., Wang W.Z., Wang Y.J., Du M.X., Liu F.L., Zhou N., Jiang C.Y., Wang C.Y., Wu L., Ma J. (2022). Metabolite profiling of human-originated Lachnospiraceae at the strain level. Imeta.

[96] Serafini S., O’Flaherty C. (2025). Novel insights into the lipid signalling in human spermatozoa. Hum. Reprod..

[97] Wu G. (2009). Amino acids: Metabolism, functions, and nutrition. Amino Acids.

[98] Eskiocak S., Gozen A.S., Taskiran A., Kilic A.S., Eskiocak M., Gulen S. (2006). Effect of psychological stress on the L-arginine-nitric oxide pathway and semen quality. Braz. J. Med. Biol. Res..

[99] Naamneh Elzenaty R., du Toit T., Flück C.E. (2022). Basics of androgen synthesis and action. Best Pract. Res. Clin. Endocrinol. Metab..

[100] Herp S., Durai Raj A.C., Salvado Silva M., Woelfel S., Stecher B. (2021). The human symbiont Mucispirillum schaedleri: Causality in health and disease. Med. Microbiol. Immunol..

[101] Bowles J., Knight D., Smith C., Wilhelm D., Richman J., Mamiya S., Yashiro K., Chawengsaksophak K., Wilson M.J., Rossant J. (2006). Retinoid Signaling Determines Germ Cell Fate in Mice. Science.

[102] Morales C., Griswold M.D. (1987). Retinol-induced stage synchronization in seminiferous tubules of the rat. Endocrinology.

[103] Bascuñán K.A., Araya M., Rodríguez J.M., Roncoroni L., Elli L., Alvarez J., Valenzuela R. (2025). Interplay of n-3 Polyunsaturated Fatty Acids, Intestinal Inflammation, and Gut Microbiota in Celiac Disease Pathogenesis. Nutrients.

[104] Collodel G., Castellini C., Lee J.C., Signorini C. (2020). Relevance of Fatty Acids to Sperm Maturation and Quality. Oxid. Med. Cell Longev..

[105] Hale B.J., Fernandez R.F., Kim S.Q., Diaz V.D., Jackson S.N., Liu L., Brenna J.T., Hermann B.P., Geyer C.B., Ellis J.M. (2019). Acyl-CoA synthetase 6 enriches seminiferous tubules with the ω-3 fatty acid docosahexaenoic acid and is required for male fertility in the mouse. J. Biol. Chem..

[106] Roqueta-Rivera M., Stroud C.K., Haschek W.M., Akare S.J., Segre M., Brush R.S., Agbaga M.P., Anderson R.E., Hess R.A., Nakamura M.T. (2010). Docosahexaenoic acid supplementation fully restores fertility and spermatogenesis in male delta-6 desaturase-null mice. J. Lipid Res..

[107] Dempsey E., Corr S.C. (2022). Lactobacillus spp. for Gastrointestinal Health: Current and Future Perspectives. Front. Immunol..

[108] Jiang Y., Bao C., Zhao X., Chen Y., Song Y., Xiao Z. (2022). Intestinal bacteria flora changes in patients with Mycoplasma pneumoniae pneumonia with or without wheezing. Sci. Rep..

[109] Li T., Tang Z., Song Y., Luo X., Zhou B., Liu X., Li F., Wang Y., Zhang L. (2026). Busulfan-Induced Male Infertility: Mechanisms, Therapeutic Interventions, and Future Directions. Andrology.

[110] Cattoni A., Nicolosi M.L., Capitoli G., Gadda A., Molinari S., Louka S., Buonsante A., Orlandi S., Salierno G., Bellani I. (2023). Pubertal attainment and Leydig cell function following pediatric hematopoietic stem cell transplantation: A three-decade longitudinal assessment. Front. Endocrinol..

[111] Panahi M., Keshavarz S., Rahmanifar F., Tamadon A., Mehrabani D., Karimaghai N., Sepehrimanesh M., Aqababa H. (2015). Busulfan induced azoospermia: Stereological evaluation of testes in rat. Vet. Res. Forum.

[112] Zhang Z.J., Cole C.G., Coyne M.J., Lin H., Dylla N., Smith R.C., Pappas T.E., Townson S.A., Laliwala N., Waligurski E. (2024). Comprehensive analyses of a large human gut Bacteroidales culture collection reveal species- and strain-level diversity and evolution. Cell Host Microbe.

[113] O’Callaghan A., van Sinderen D. (2016). Bifidobacteria and Their Role as Members of the Human Gut Microbiota. Front. Microbiol..

[114] Rautava S., Walker W.A., Lu L. (2016). Hydrocortisone-induced anti-inflammatory effects in immature human enterocytes depend on the timing of exposure. Am. J. Physiol. Gastrointest. Liver Physiol..

[115] Liu Y., Myojin T., Li K., Kurita A., Seto M., Motoyama A., Liu X., Satoh A., Munemasa S., Murata Y. (2022). A Major Intestinal Catabolite of Quercetin Glycosides, 3-Hydroxyphenylacetic Acid, Protects the Hepatocytes from the Acetaldehyde-Induced Cytotoxicity through the Enhancement of the Total Aldehyde Dehydrogenase Activity. Int. J. Mol. Sci..

[116] Imai H., Hakkaku N., Iwamoto R., Suzuki J., Suzuki T., Tajima Y., Konishi K., Minami S., Ichinose S., Ishizaka K. (2009). Depletion of selenoprotein GPx4 in spermatocytes causes male infertility in mice. J. Biol. Chem..

[117] Liu S., Cao R., Liu L., Lv Y., Qi X., Yuan Z., Fan X., Yu C., Guan Q. (2022). Correlation Between Gut Microbiota and Testosterone in Male Patients With Type 2 Diabetes Mellitus. Front. Endocrinol..

[118] Hatta M.N.A., Mohamad Hanif E.A., Chin S.F., Low T.Y., Neoh H.M. (2023). Parvimonas micra infection enhances proliferation, wound healing, and inflammation of a colorectal cancer cell line. Biosci. Rep..

[119] Sciavilla P., Strati F., Di Paola M., Modesto M., Vitali F., Cavalieri D., Prati G.M., Di Vito M., Aragona G., De Filippo C. (2021). Gut microbiota profiles and characterization of cultivable fungal isolates in IBS patients. Appl. Microbiol. Biotechnol..

[120] Li J., Li Y., Zhou Y., Wang C., Wu B., Wan J. (2018). Actinomyces and Alimentary Tract Diseases: A Review of Its Biological Functions and Pathology. Biomed. Res. Int..

[121] Huang C., Chen J., Wang J., Zhou H., Lu Y., Lou L., Zheng J., Tian L., Wang X., Cao Z. (2017). Dysbiosis of Intestinal Microbiota and Decreased Antimicrobial Peptide Level in Paneth Cells during Hypertriglyceridemia-Related Acute Necrotizing Pancreatitis in Rats. Front. Microbiol..

[122] Matsushita M., Fujita K., Motooka D., Hatano K., Hata J., Nishimoto M., Banno E., Takezawa K., Fukuhara S., Kiuchi H. (2022). Firmicutes in Gut Microbiota Correlate with Blood Testosterone Levels in Elderly Men. World J. Men’s Health.

[123] Liu C.Y., Chou Y.C., Chao J.C., Hsu C.Y., Cha T.L., Tsao C.W. (2015). The Association between Dietary Patterns and Semen Quality in a General Asian Population of 7282 Males. PLoS ONE.

[124] Jensen T.K., Heitmann B.L., Jensen M.B., Halldorsson T.I., Andersson A.-M., Skakkebæk N.E., Joensen U.N., Lauritsen M.P., Christiansen P., Dalgård C. (2013). High dietary intake of saturated fat is associated with reduced semen quality among 701 young Danish men from the general population123. Am. J. Clin. Nutr..

[125] Attaman J.A., Toth T.L., Furtado J., Campos H., Hauser R., Chavarro J.E. (2012). Dietary fat and semen quality among men attending a fertility clinic. Hum. Reprod..

[126] Mendiola J., Torres-Cantero A.M., Moreno-Grau J.M., Ten J., Roca M., Moreno-Grau S., Bernabeu R. (2009). Food intake and its relationship with semen quality: A case-control study. Fertil. Steril..

[127] Wang Z.-h., Kang Y.-f. (2025). Gut microbiota and male fertility: A two-sample Mendelian randomization study. Medicine.

[128] Cao T., Wang S., Pan Y., Guo F., Wu B., Zhang Y., Wang Y., Tian J., Xing Q., Liu X. (2023). Characterization of the semen, gut, and urine microbiota in patients with different semen abnormalities. Front. Microbiol..

[129] Stanhope K.L., Schwarz J.M., Keim N.L., Griffen S.C., Bremer A.A., Graham J.L., Hatcher B., Cox C.L., Dyachenko A., Zhang W. (2009). Consuming fructose-sweetened, not glucose-sweetened, beverages increases visceral adiposity and lipids and decreases insulin sensitivity in overweight/obese humans. J. Clin. Investig..

[130] Park K., Gross M., Lee D.-H., Holvoet P., Himes J.H., Shikany J.M., Jacobs D.R. (2009). Oxidative Stress and Insulin Resistance: The Coronary Artery Risk Development in Young Adults study. Diabetes Care.

[131] Dena S.M., Adeleye A.O., Mohlala K., Langa B.C., Opuwari C.S. (2025). The Impact of Diabetes Mellitus-Related Oxidative Stress on Male Fertility: A Review. J. Diabetes.

[132] Ahmed M.G., Ibrahim M.E.-D., El Sayed H.R., Ahmed S.M. (2021). Short term chronic toxicity of tributyltin on the testes of adult albino rats and the possible protective role of omega-3. Hum. Exp. Toxicol..

[133] Hasan M.M., El-Shal A.S., Mackawy A.M.H., Ibrahim E.M., Abdelghany E., Saeed A.A., El-Gendy J. (2020). Ameliorative effect of combined low dose of Pioglitazone and omega-3 on spermatogenesis and steroidogenesis in diabetic rats. J. Cell Biochem..

[134] Ge H., Cai Z., Chai J., Liu J., Liu B., Yu Y., Liu J., Zhang T. (2021). Egg white peptides ameliorate dextran sulfate sodium-induced acute colitis symptoms by inhibiting the production of pro-inflammatory cytokines and modulation of gut microbiota composition. Food Chem..

[135] Louca P., Nogal A., Wells P.M., Asnicar F., Wolf J., Steves C.J., Spector T.D., Segata N., Berry S.E., Valdes A.M. (2021). Gut microbiome diversity and composition is associated with hypertension in women. J. Hypertens..

[136] Xue L., He J., Gao N., Lu X., Li M., Wu X., Liu Z., Jin Y., Liu J., Xu J. (2017). Probiotics may delay the progression of nonalcoholic fatty liver disease by restoring the gut microbiota structure and improving intestinal endotoxemia. Sci. Rep..

[137] Kurata S., Hiradate Y., Umezu K., Hara K., Tanemura K. (2019). Capacitation of mouse sperm is modulated by gamma-aminobutyric acid (GABA) concentration. J. Reprod. Dev..

[138] Cai H., Cao X., Qin D., Liu Y., Liu Y., Hua J., Peng S. (2022). Gut microbiota supports male reproduction via nutrition, immunity, and signaling. Front. Microbiol..

[139] Liu C., Huang S., Wu Z., Li T., Li N., Zhang B., Han D., Wang S., Zhao J., Wang J. (2021). Cohousing-mediated microbiota transfer from milk bioactive components-dosed mice ameliorate colitis by remodeling colonic mucus barrier and lamina propria macrophages. Gut Microbes.

[140] Matchado M.S., Rühlemann M., Reitmeier S., Kacprowski T., Frost F., Haller D., Baumbach J., List M. (2024). On the limits of 16S rRNA gene-based metagenome prediction and functional profiling. Microb. Genom..

[141] de Kretser D.M. (2010). Determinants of male health: The interaction of biological and social factors. Asian J. Androl..

